# The role of oxygen vacancies in the sensing properties of Ni substituted SnO_2_ microspheres†

**DOI:** 10.1039/c8ra05679j

**Published:** 2018-09-24

**Authors:** Jianwu Sun, Guilin Yin, Ting Cai, Weiwei Yu, Fang Peng, Yan Sun, Fang Zhang, Jing Lu, Meiying Ge, Dannong He

**Affiliations:** School of Material Science and Engineering, Shanghai Jiao Tong University No. 800 Dongchuan Road Shanghai 200240 PR China; National Engineering Research Center for Nanotechnology No. 28 East Jiang Chuan Road Shanghai 200241 PR China meiyingge@163.com; National Laboratory for Infrared Physics, Shanghai Institute of Technical Physics, Chinese Academy of Sciences No. 500 Yutian Road Shanghai 200083 PR China

## Abstract

The influence of Ni doping in SnO_2_ microspheres was investigated in this study. SnO_2_ was doped with different amounts of Ni using a simple dipping method. The doped SnO_2_ structure, which was confirmed from X-ray photoelectron (XPS) and photoluminescence (PL) spectroscopies, was shown to possess distinctly more oxygen vacancies. Oxygen vacancies were found to be responsible for the surface adsorption of oxygen, as shown in the O 1s XPS spectrum and O_2_-TPD (temperature programmed desorption) measurements which can enhance the sensitivity of materials. According to the gas sensing properties, Ni-doped SnO_2_ was enhanced towards ethanol and showed excellent stability at the operating temperature. At 1 ppm of ethanol vapor, the response value of Ni substituted SnO_2_ was about 3 times that of pristine SnO_2_ microspheres. This research reveals a notable perspective for the design of sensing materials in terms of Ni substitutional doping.

## Abstract 2

The influence of Ni doping in SnO_2_ microspheres was investigated in this study.

## Graphical abstract

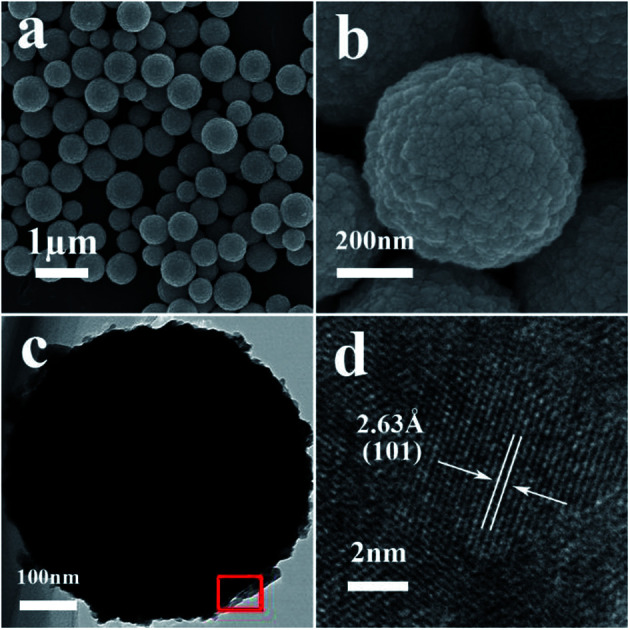

## Introduction

As a typical wide band gap n-type semiconductor, SnO_2_ has been studied extensively as a gas sensor due to its excellent chemical and electrical properties.^1–3^ Over the past few years, many different types of structures and morphologies of SnO_2_ have been fabricated *via* various methods,^4–6^ because it was found that the grain size, surface state and nanostructure composition have a great effect on the gas sensing process.^7,8^ In order to further improve the sensing performance, especially the sensitivity, many doped,^9–11^ loaded,^12,13^ or decorated^14,15^ semiconductors have been synthesized. The sensing mechanism of SnO_2_ is associated with the adsorption of surface oxygen, in which the grain size, surface state and composition have a great effect on the gas sensing process. It is well known that oxygen vacancies play an essential role in determining the sensing performance of sensors. Recent research has revealed that doping is a simple and effective method that can be used to promote the intrinsic poor sensitivity and selectivity of SnO_2_.^16,17^

Numerous metal oxide^5,18^ and noble metal^19,20^ dopants have been introduced into SnO_2_ to improve its sensing properties. Among the various different dopants, NiO is known as a potential catalyst for enhancing the sensitivity.^9^ Besides this, in Ni-doped SnO_2_, the Ni^2+^ dopant can occupy the Sn^4+^ ion sites in the matrix of SnO_2_ on account of the similarity in the radii of Ni^2+^ (0.069 nm) and Sn^4+^ (0.071 nm),^21^ which leads to an increase in the number of oxygen vacancies in the material, according to the following defect reaction (eqn (1)):^22^1
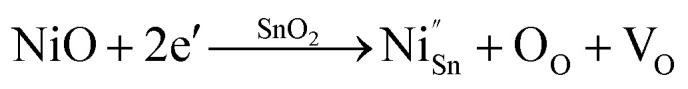


Oxygen molecules in the atmosphere tend to adsorb onto these defective oxygen vacancies, forming an electron depletion layer near the SnO_2_ surface, Therefore, substitutional doping of Ni in SnO_2_ might activate the surface of SnO_2_ and enhance its sensing properties. Gu *et al.* prepared porous Ni-doped SnO_2_ microspheres and microcubes *via* a facile chemical solution route, which were found to exhibit enhanced sensing properties towards toxic VOCs, such as formaldehyde, ethanol, benzene, methanol, acetone, and toluene.^23^ Ni doped SnO_2_ materials with different Ni concentrations were synthesized by Lin *et al.* and sensors based on 2 mol% Ni doped SnO_2_ and 4 mol% Ni doped SnO_2_ showed ultrahigh responses to *n*-butanol and formaldehyde with good selectivity, respectively.^2^ Despite many studies that have shown that surface adsorbed oxygen plays an important role in the gas sensing process, few studies have focused on the origin of the oxygen vacancies and surface adsorbed oxygen, which is important to further understand the sensing mechanism of doped semiconductor-based gas sensors.

In this work, we design a facile dipping process that can be used for the substitutional doping of Ni in SnO_2_ to improve the sensitivity of pure SnO_2_. The physicochemical properties of the samples were characterized by powder X-ray diffraction (PXRD), X-ray photoelectron spectroscopy (XPS), photoluminescence (PL) spectroscopy and O_2_-temperature programmed desorption (O_2_-TPD). It was postulated that the amount of surface adsorbed oxygen introduced by oxygen vacancies in the Ni-doped SnO_2_ sensor mainly contributes to the enhanced sensitivity and selectivity of the sensor to ethanol gas. Research on the relationship between oxygen vacancies and the surface adsorbed oxygen of metal oxide semiconductors is important and interesting for the rational design of sensing materials and structures.

## Experimental

### Synthesis

All chemicals (A.R. grade) used in the experiment were purchased from Shanghai Chemical Co. and used directly without further purification.

### Preparation of the pure SnO_2_ microspheres

Microspheres of SnO_2_ were prepared *via* a facile hydrothermal method. In a typical procedure, 0.35 g of SnCl_4_·5H_2_O was dissolved in 50 mL of ethanol and 5 mL of distilled water followed by the addition of 1 mL of concentrated hydrochloric acid (mass fraction 36–38%). Then, the mixture was ultrasonicated for 30 min to obtain a homogeneous solution. After that, the above mixture was transferred into a 100 mL stainless autoclave lined with a Teflon vessel and kept at a constant temperature of 200 °C for 24 h. Afterwards, the resulting white precipitate was collected by centrifugation and washed several times, alternating between deionized water and ethanol. The sample was then dried at 60 °C for 12 h and calcined at 400 °C for 2 h. The final sample is referred to as S0.

### Preparation of the Ni-doped SnO_2_ microspheres

The Ni-doped SnO_2_ microspheres were synthesized through a dipping method, which can be described briefly as follows: 0.17 g of the obtained SnO_2_ powder mentioned above was dispersed in 25 mL of an aqueous solution of NiCl_2_ (0.5 mol L^−1^, 2 mol L^−1^ and 5 mol L^−1^, denoted as S1, S2 and S3, respectively) under continuous magnetic stirring. After 24 h of stirring, the precipitate was collected by centrifugation and dried directly at 60 °C for 24 h. Eventually, the Ni-doped SnO_2_ microspheres were obtained after calcining at 600 °C for 3 h.

### Characterization

The morphology and structure of the as-prepared samples were observed by employing a field-emission scanning electron microscope (FESEM, Hitachi S-4800) and transmission electron microscope (TEM, JEM-2100F JEOL electron microscope). PXRD measurements were carried out on a D/max-2600PC equipped with a Cu Kα radiation source (*λ* = 1.5406 Å). XPS measurements were performed on a Kratos Axis Ultra DLD spectrometer with an Al X-ray monochromator. PL spectra were recorded by a fluorescence spectrometer (Hitachi F-7000). O_2_-TPD measurements were carried out in a U-shaped quartz tube and the desorption signal of oxygen was recorded with an online mass spectrometer apparatus (HIDEN QIC-20).

### Fabrication and testing of the gas sensor

Typically, a certain amount of synthesized product was mixed with several drops of ethanol in an agate mortar to form a homogeneous paste, which was then coated onto the outer surface of a ceramic tube positioned with a pair of Au electrodes and four Pt wires on both ends of the tube. Then a Ni–Cr alloy coil (10 Ω) was inserted into the tube as a heater to tune the operating temperature. Gas sensing tests were performed on a commercial HW-30A gas sensing measurement system (Hanwei Electronics Co. Ltd., Henan, PR China), which is a static system that uses atmospheric air as the interference gas. Before testing, the sensors were aged at the working temperature for several days to improve the repeatability and stability. The sensor response (*S*) is defined as *R*_a_/*R*_g_, where *R*_a_ and *R*_g_ are the electrical resistance of the sensor in air and in the target gas, respectively.

## Results and discussion

### Microstructure

**Fig. 1:**
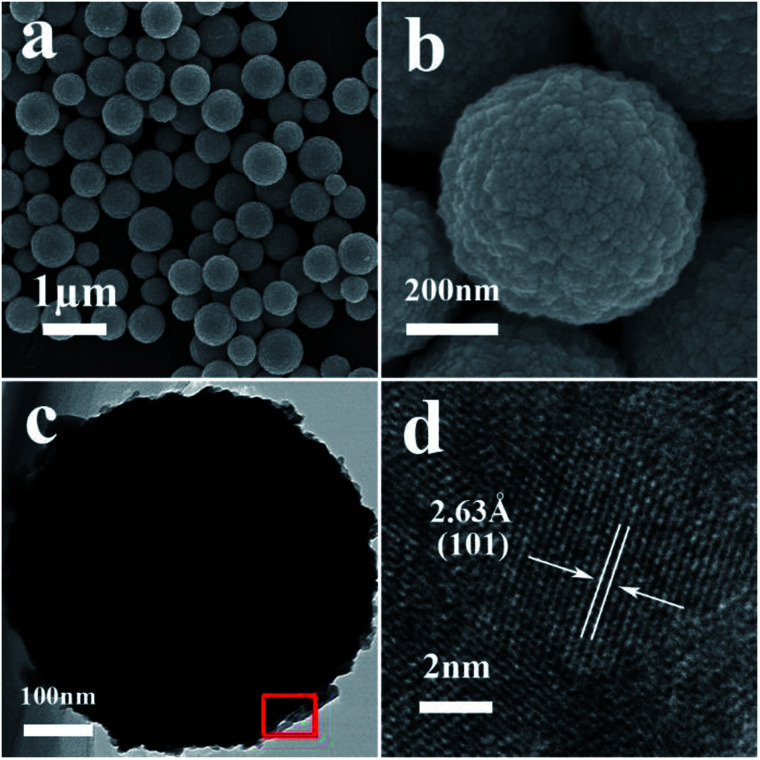
(a) A typical FESEM image of the pure SnO_2_ microspheres, (b) high-magnification FESEM image, (c) a typical TEM image of a pure SnO_2_ microsphere, and (d) a HRTEM image obtained from the marked fringe of (c).

**Fig. 2:**
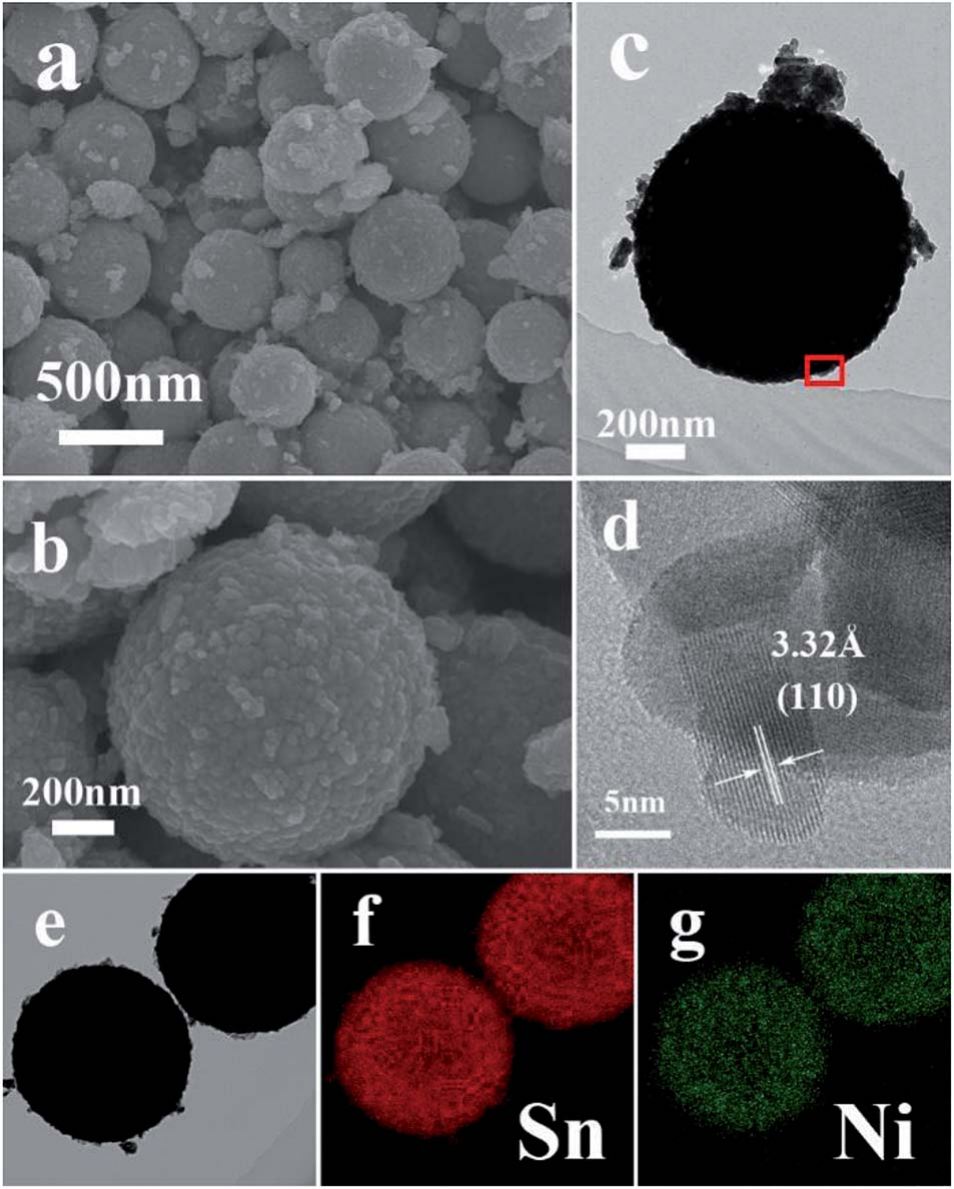
(a and b) Typical FESEM images of S1, (c) typical TEM image of S1, (d) high-resolution TEM image of the marked fringe of (c), (e) TEM image and (f and g) the corresponding elemental mapping images of Sn and Ni in (e).

Typical FESEM and TEM images of the as-synthesized samples are presented in Fig. 1 and 2. The low-magnification FESEM image (Fig. 1a) shows an overall view of the pure SnO_2_ microspheres, which are composed of numerous spherical architectures with a uniform size, without the presence of other morphologies. The magnified image in Fig. 1b suggests that the diameter of the obtained SnO_2_ microspheres is around 400 nm and that the surface is composed of irregular nanoparticles. Fig. 1d shows the HRTEM image obtained from the marked fringe of a typical TEM image, shown in Fig. 1c, and the resolved fringes separated by 2.63 Å correspond to the (101) lattice planes of tetragonal SnO_2_.

After the slight doping of Ni in the SnO_2_ microspheres (S1), a typical low-magnification FESEM image was recorded, shown in Fig. 2a, from which the microspheres can still be observed with a few particles around them. Fig. 2b–c shows the detailed morphology of a single Ni-doped SnO_2_ microsphere. The resolved fringes observed from the HRTEM image (Fig. 2d) indicate that the lattice spacing of adjacent lattice fringes is around 3.32 Å, which corresponds to the (110) lattice planes of tetragonal SnO_2_. The elemental mapping images of Sn and Ni displayed in Fig. 2e–g suggest that the Ni atoms are uniformly dispersed in the SnO_2_ microspheres.

As the concentration of the dipping solution increased, more Ni^2+^ was absorbed on the surface of the microspheres. As shown in Fig. S1 and S2,† more fragments gathered around the microspheres. The HRTEM and elemental mapping images show that the fragments were composed of the NiO phase as a result of the heat treatment, and that the excess Ni^2+^ tended to nucleate and crystallize instead of substituting for Sn^4+^ in the lattice.

### Phase Structure and Composition

**Fig. 3:**
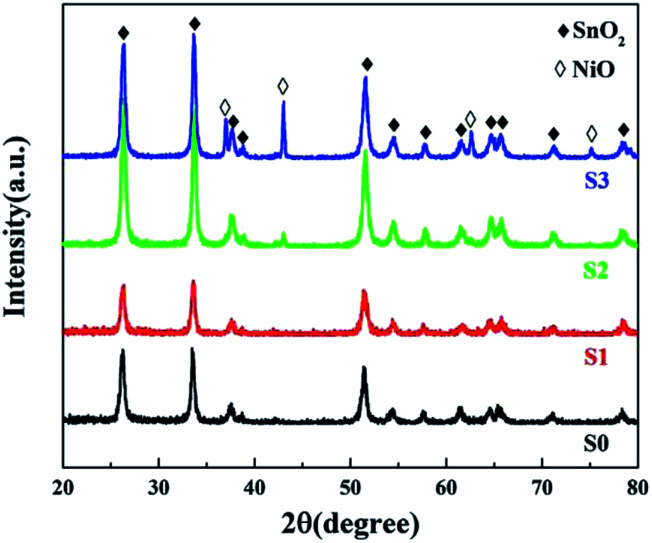
PXRD patterns of pure and Ni-doped SnO_2_.

As shown in Fig. 3, all of the diffraction peaks for both S0 and S1 can be indexed to single-phase tetragonal structured SnO_2_ (JCPDS no. 41-1445). The phase structure remained nearly unchanged for S1 and no NiO phase was detected. Furthermore, no evident shift in the peaks was observed after Ni doping, due to the similarity in the ionic radii of Sn^4+^ (0.071 nm) and Ni^2+^ (0.069 nm).^24^ As the NiCl_2_ concentration increased to 2 mol L^−1^, extra peaks emerged that could be indexed as NiO (JCPDS no. 47-1049), indicating that the excess Ni^2+^ ions could not access the lattice structure of SnO_2_ and instead formed a NiO phase. The NiO peaks were further enhanced in the spectrum of S3 indicating that slight doping with Ni^2+^ leads to substitution of Sn^4+^.

**Fig. 4:**
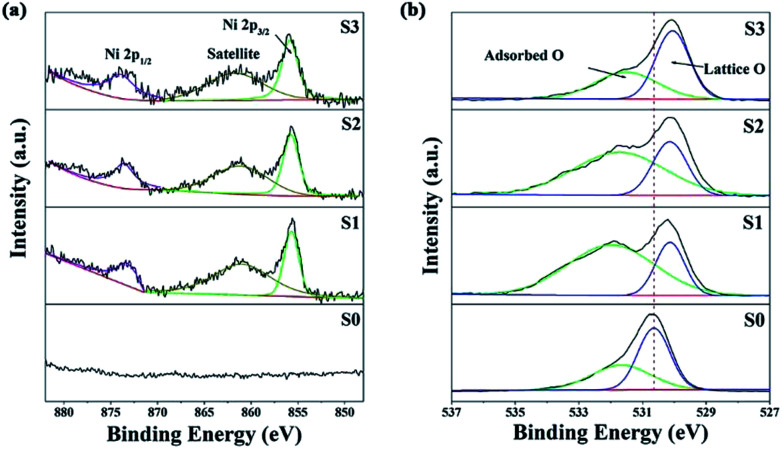
The Ni 2p (a) and O 1s (b) XPS spectra.

XPS analysis was carried out to further characterize the as-prepared samples and illustrate their surface compositions and chemical states. In the full range spectra (Fig. S4a†), Sn and O were found to be the predominant elements in all the samples, and Ni was also detected in the Ni-doped samples. No impurity elements, except for C, were observed. The binding energy for the C 1s peak at 284.8 eV was used as a reference for the energy calibration. For pure SnO_2_, the Sn 3d spectra (Fig. S4b†) has two strong peaks at a binding energy of 495.2 and 486.8 eV with a spin–orbit splitting of 8.4 eV, corresponding to Sn 3d_5/2_ and 3d_3/2_, respectively.^25^ These results were in good agreement with those in literature reports, indicating the presence of Sn with a valence of +4.^1^ With an increase in the concentration of the dipping solution, the Sn 3d_5/2_ and 3d_3/2_ peaks shifted towards a lower binding energy, implying a decrease in the Sn oxidation state.^26^Fig. 4a shows the Ni 2p high resolution XPS spectra. The peaks at 860 and 855.6 eV can be attributed to Ni 2p_3/2_, and the 873.5 eV peaks belong Ni 2p_1/2_.^27^ Furthermore, the quantification of peaks revealed that the Ni atom percentage (at%) was ∼0.96%, ∼3.82% and ∼4.99% for S1, S2 and S3, respectively. The O 1s peak of SnO_2_ in Fig. 4b is asymmetric and it could be Gaussian divided into two components: a low binding energy (530.6 eV) component typical of lattice O^2−^ ions, and a high binding energy (531.7 eV) component corresponding to adsorbed O^−^ and O^2−^ on the surface of the microspheres. Similar to the Sn 3d spectra, the O 1s peak of lattice O^2−^ ions also shifted towards a lower binding energy after Ni doping. This might be attributed to the lower electronegativity of Ni to that of Sn, which resulted in a larger screening effect for the O atoms.^24^ The shift in the Sn 3d and O 1s peaks also suggested the formation of a Ni-doped SnO_2_ solid solution rather than a NiO/SnO_2_ composite.^21^ The relative percentages of the surface adsorbed oxygen were about 40.2%, 74.2%, 68.6% and 50.7% for S0, S1, S2 and S3, respectively. These results reveal that the slightly Ni-doped SnO_2_ microspheres (S1) had a greater ability to adsorb ionized oxygen species.

**Fig. 5:**
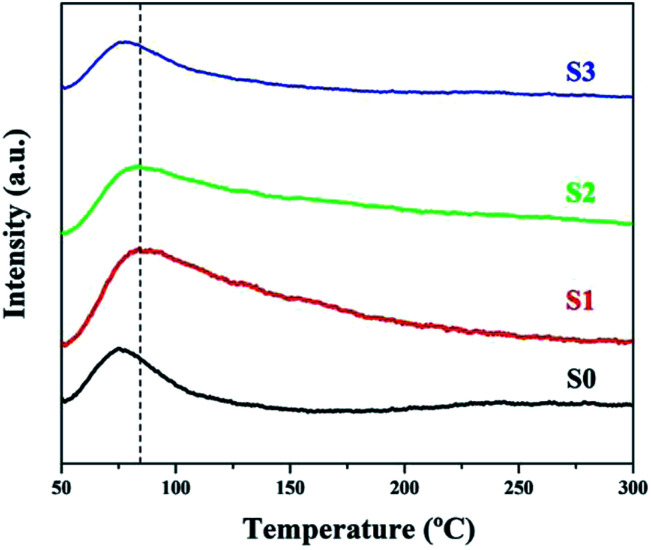
O_2_-TPD profiles of all of the samples.

O_2_-TPD measurements are an effective way of determining the amount of adsorbed oxygen on the material surface, which is a crucial factor in the sensitivity of the gas sensing process.^28^ As shown in Fig. 5, the desorption of oxygen at a very low temperature (<150 °C) for both samples can be ascribed to physically adsorbed oxygen and/or chemically adsorbed oxygen.^29^ For the Ni-doped samples, the desorption peaks broadened at high temperatures, especially for S1, indicating an increase in the chemically adsorbed oxygen species, because the physically adsorbed oxygen is more easily desorbed than chemically adsorbed oxygen as the temperature rises. Also, there was an obvious increase in the desorption peak intensity for S1, reflecting the high amount of surface adsorbed oxygen. It is hypothesized that this was due to the high number of oxygen vacancies introduced by Ni doping.

**Fig. 6:**
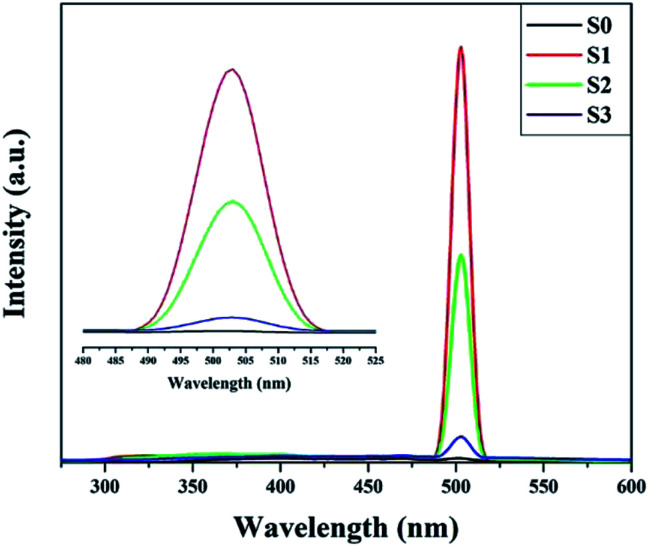
Photoluminescence spectra of pure and Ni-doped SnO_2_.

In order to clarify the origin of the increase in the surface adsorbed oxygen, we performed photoluminescence spectroscopy, which can be used to investigate surface defects, impurities and energy bands. Fig. 6 shows the room temperature PL spectra for SnO_2_ microspheres with an excitation wavelength of 250 nm. A broad emission band was observed between 380 and 410 nm for both samples. This band was attributed to the free exciton recombination from the conduction band edge to the valence band edge.^26^ The blue green emission bands centered at 452 and 470 nm might correspond to the formation of doubly charged oxygen vacancies 
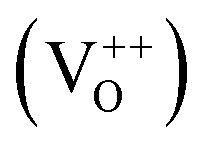
.^30^ The green emission band at 503 nm was thought to originate from singly charged oxygen vacancies 
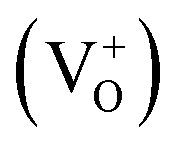
. The intensity of this emission band increased drastically for S1, while for S2 and S3, the intensity decreased, which might be due to the variation in the density of oxygen vacancies.^31^ For the Ni-doped samples, the concentration of the oxygen vacancies increased according to the defect reaction (eqn (1)) during the Ni doping. However, as the concentration of the dipping solution increased, the excess Ni^2+^ tended to nucleate and crystallize instead of substitute for Sn^4+^ in the lattice. The slightly doped SnO_2_ microspheres (S1) had the highest amount of surface adsorbed oxygen, although the Ni content of S1 was less than that of S2 and S3.

### Gas-sensing properties

**Fig. 7:**
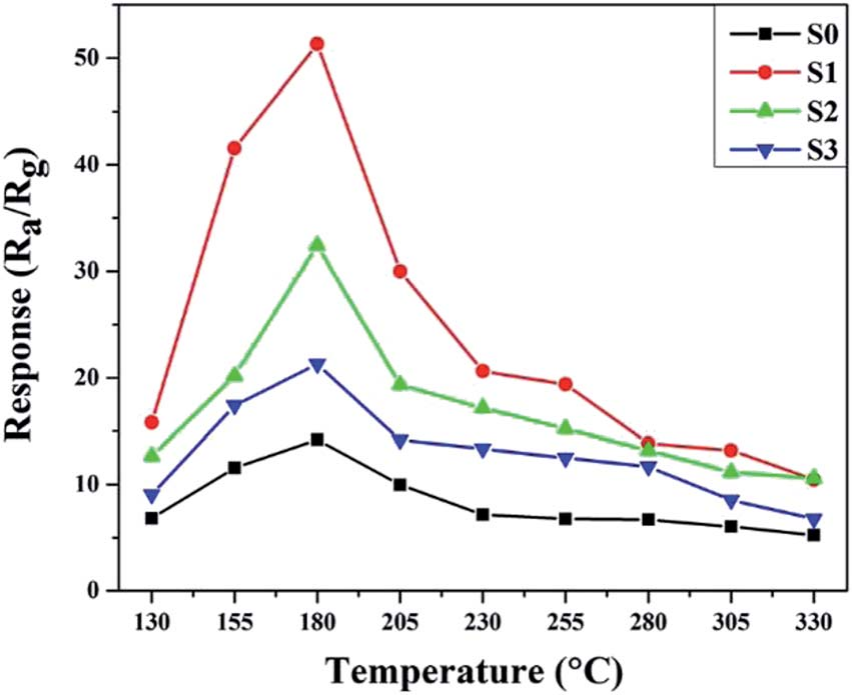
The sample responses to 100 ppm of ethanol at various temperatures.

The operating temperature is an important factor for semiconductor gas sensors, as has been previously described in the literature. Therefore, the function of the gas response to the operating temperatures of sensors based on pure and Ni-doped SnO_2_ microspheres to 100 ppm ethanol was investigated and the results are shown in Fig. 7. The sensing responses were observed to increase upon an increase in the operating temperature from 130 to 180 °C. This result is due to an increase in the presence of surface adsorbed oxygen in the form of O_2_^−^, O^2−^ and O^−^ at higher temperatures and because more ethanol molecules react with the surface of the sensing material, decreasing the resistance of the sample.^2,32^ Subsequently, the response of the sensor based on S1 reached a maximum value of 51.3 for 100 ppm of ethanol, which was 3.62 times higher than the sensor based on S0 at the same operating temperature of 180 °C. After the maximum value, the response decreased upon a further increase in the operating temperature, which might be caused by the adsorption of ethanol molecules being lower than the desorption at such a high temperature.^33,34^ All other sensing tests were performed at the optimum operating temperature.

**Fig. 8:**
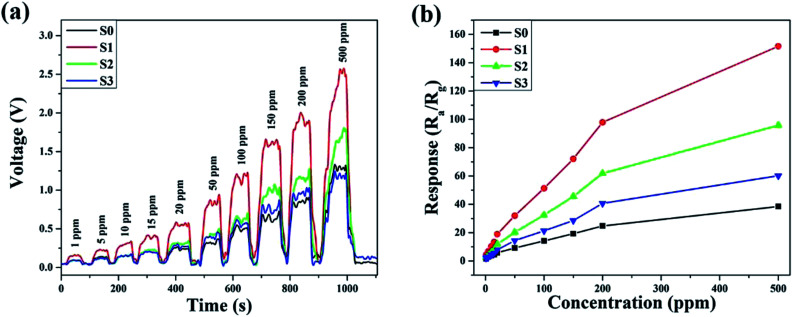
Dynamic response–recovery curves of the samples to ethanol at 180 °C.

The dynamic response–recovery curves towards 1–500 ppm of ethanol at 180 °C were measured and the results are shown in Fig. 8. In Fig. 8a, the electrical resistance of both samples can be seen to drastically increase with time as the ethanol was introduced and then rapidly returned to the baseline after the ethanol was exhausted in the testing system. Meanwhile, the two gas sensors exhibited excellent response and recovery performances to different concentrations of ethanol. As shown in Fig. 8b, at a low ethanol concentration (1 ppm), the relative responses of S0, S1, S2 and S3 were approximately 1.49, 4.43, 2.8 and 2.01, respectively. The response amplitudes of the sensors increased sharply upon an increase in the ethanol concentration, and it could be clearly observed that the response significantly enhanced after Ni doping. The enhanced sensitivity is probably due to the increase in the amount of surface adsorbed oxygen.

**Fig. 9:**
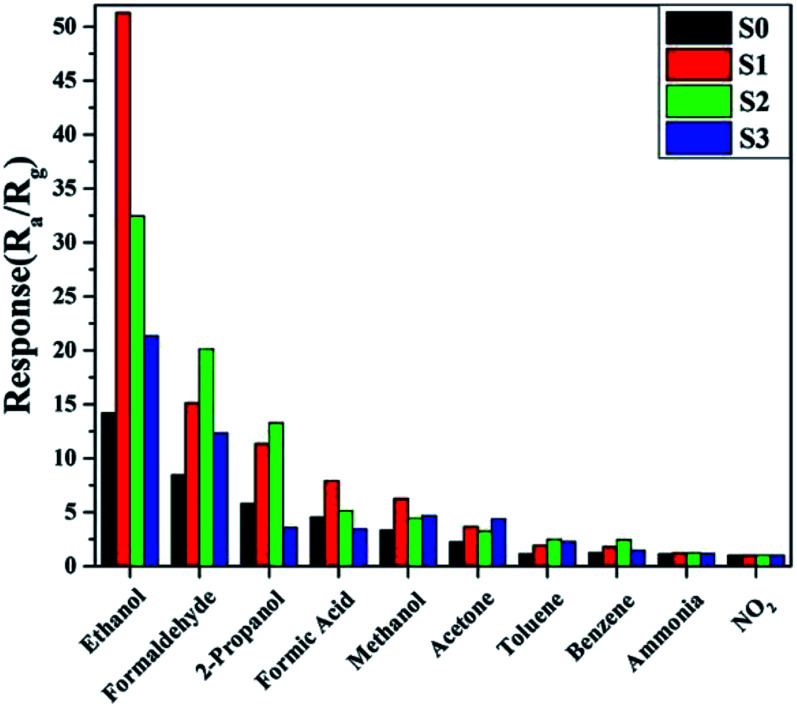
Sample responses to 100 ppm of various testing gases at 180 °C.

Furthermore, the selectivity of all of the samples to 100 ppm of different gases was tested at the same temperature of 180 °C and the results are shown in Fig. 9. It is obvious that the sensor based on S1 displayed enhanced responses and good selectivity to the tested gases, and the response to ethanol was observed to be higher than for other gases. This indicated that the slight doping of Ni in SnO_2_ not only increased the sensitivity, but also improved the selectivity.

**Fig. 10:**
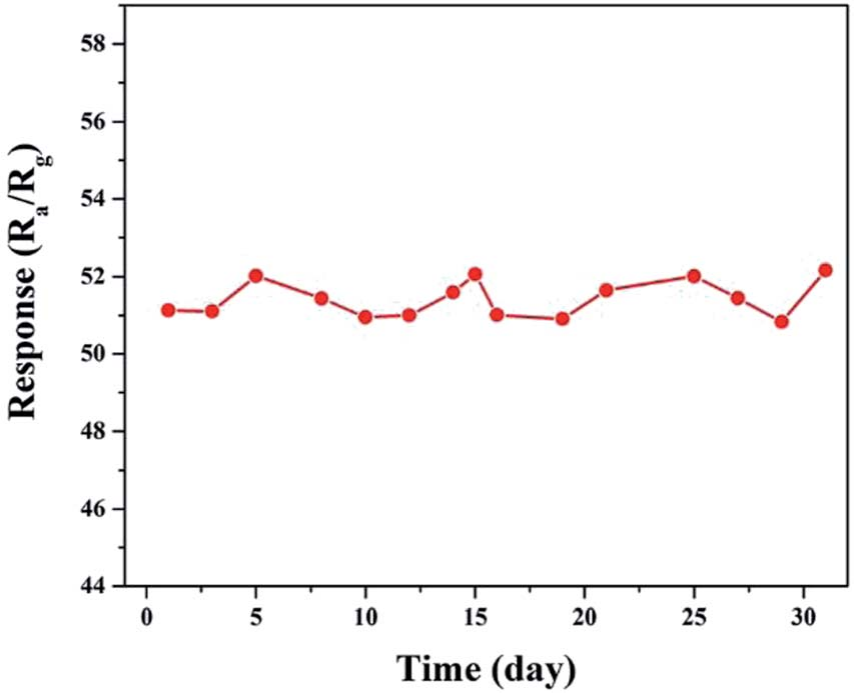
Long-term tests on the response to 100 ppm of ethanol at 180 °C.

The long-term and humidity stability are also important parameters for the practical application of a gas sensor. To illustrate this aspect, a 30 day experiment on an S1-based gas sensor was performed. As shown in Fig. 10, the change in the amplitude of the responses of the sensor to 100 ppm of ethanol during the 30 days was controlled within a small range of 50.5–52.5. In addition, the responses of the sensors remained almost unchanged under different relative humidity values at 180 °C towards 100 ppm of ethanol, as shown in Fig. S5.† The excellent stability of the slightly Ni-doped SnO_2_ sensor (S1), in combination with its high sensitivity and selectivity, makes it a promising material for use in practical applications.

### Gas-sensing mechanism

For most n-type semiconductor gas sensors, the most widely accepted sensing mechanism is based on the variation of the depletion layer in the sensing material due to the adsorption and desorption of gas molecules.^24^ When the sensing material is exposed to ambient air, oxygen molecules adsorb on the surface of the microspheres. These adsorbed oxygen molecules transform into surface-adsorbed ionized oxygen species (O_2_^−^, O^2−^, O^−^) by capturing the free electrons from the conduction band of SnO_2_, which results in an increase in the resistance of the gas sensors. When the Ni-doped SnO_2_ gas sensors are exposed to a reducing gas such as ethanol, the surface adsorbed oxygen reacts with the ethanol molecules on the surface of the microspheres. Consequently, the electrons are released back into the conduction band, leading to a thinner depletion layer and a decrease in the resistance of the gas sensors.^35^ Based on this mechanism, the sensing performance of the sensor depends on the ability of the sensing materials to adsorb and ionize oxygen species.

For Ni-doped samples, the electron density decreases while the concentration of oxygen vacancies increases according to the defect reaction (eqn (1)) during the Ni doping. As oxygen vacancies are normally active sites for surface reactions, Ni-doping can increase the amount of oxygen species that adsorb and ionize on the surface of SnO_2_. As the excess Ni^2+^ in S2 and S3 prefers to nucleate and crystallize, S1 was found to have the highest amount of Ni^2+^ substitution, although the Ni doping content in S1 is less than that in S2 and S3. This decreases the surface electron density, resulting in a thickening of the electron depletion layer and an increase in the resistance. The baseline resistances of the sensors are 6.23, 28.14, 4.09 and 6.94 MΩ for S0, S1, S2 and S3, respectively. When S1 is exposed to ethanol gas, numerous electrons are released from the surface adsorbed oxygen, inducing a strong shrinkage in the depletion layer. Therefore, a considerable change in the depletion region was observed, giving rise to the excellent sensitivity of the slightly Ni-doped SnO_2_ sensor.

## Conclusions

In summary, slightly Ni doped SnO_2_ microspheres, synthesized *via* a dipping method, were investigated as high-performance gas sensing materials for the detection of ethanol. XRD and XPS measurements showed that Ni was doped in the SnO_2_ crystal lattice. PL, XPS and O_2_-TPD characterization confirmed that the amount of the surface adsorbed oxygen significantly increased due to the higher content of oxygen vacancies introduced by Ni doping, and that the adsorbed oxygen transformed into surface-adsorbed ionized oxygen species (O_2_^−^, O^2−^, O^−^), which enhanced the gas sensing properties of pure SnO_2_. The gas sensing tests showed that the sensor response to ethanol increased after Ni doping compared to the sensing with just pure SnO_2_ at 180 °C. In addition, the sensors based on Ni-doped SnO_2_ also exhibited good stability and selectivity.

## Conflicts of interest

There are no conflicts to declare.

## Supplementary Material

RA-008-C8RA05679J-s001
