# Supplementary material for: The role of oxygen vacancies in the sensing properties of Ni substituted SnO2 microspheres
Source: RSC Adv. 2018 Sep 24;8(58):33080–6. doi: 10.1039/c8ra05679j (PMC9086386; doi:10.1039/c8ra05679j)
Supplement: RA-008-C8RA05679J-s001 [file RA-008-C8RA05679J-s001.pdf]

Electronic Supplementary Material (ESI) for RSC Advances.
This journal is © The Royal Society of Chemistry 2018

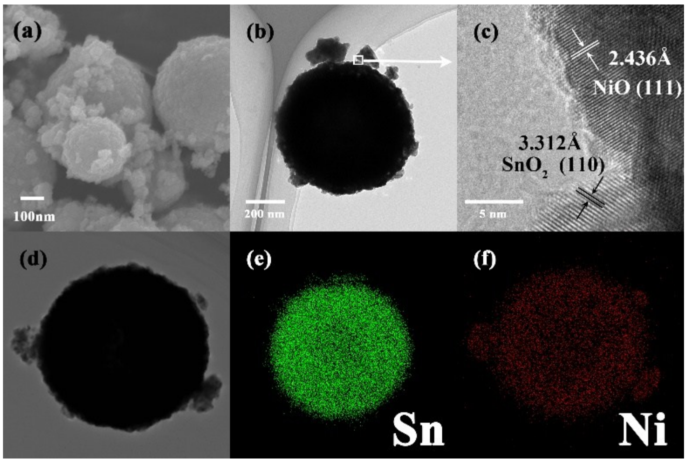

Fig.S1 (a) Typical FESEM image of S2; (b) typical TEM images of S2; (c) HRTEM image obtained from the marked fringe of (b); (d) TEM image and (e, f) the corresponding elemental mapping images of Sn and Ni.

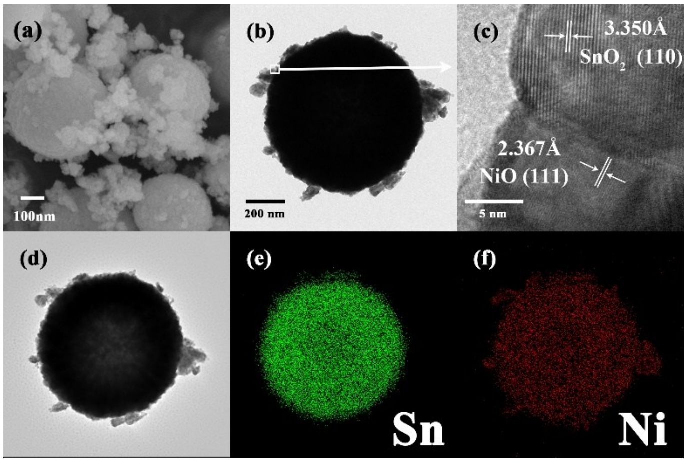

Fig.S2 (a) Typical FESEM image of S3; (b) typical TEM images of S3; (c) HRTEM image obtained from the marked fringe of (b); (d) TEM image and (e, f) the corresponding elemental mapping images of Sn and Ni.

S1

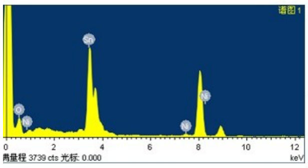

Element	Weight Percentage (wt%)	Atom Percentage (at%)
O K	7.12	35.97
Ni K	1.09	1.5
Sn L	91.79	62.53
Total	100	

S2

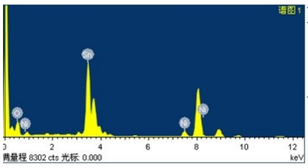

Element	Weight Percentage (wt%)	Atom Percentage (at%)
O K	6.53	33.35
Ni K	3.27	4.55
Sn L	90.2	62.1
Total	100	

S3

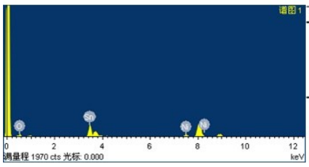

Element	Weight Percentage (wt%)	Atom Percentage (at%)
O K	7.12	35.24
Ni K	4.1	5.54
Sn L	88.78	59.23
Total	100	

Fig.S3 EDS element content of S1, S2 and S3.

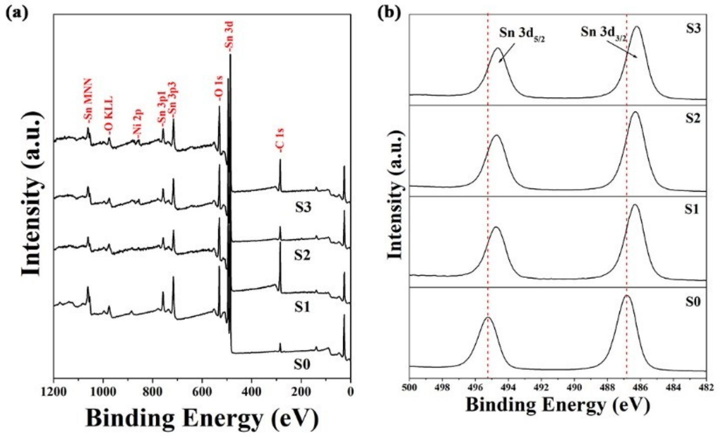

Fig.S4 XPS spectra of full spectra (a) and Sn 3d (b).

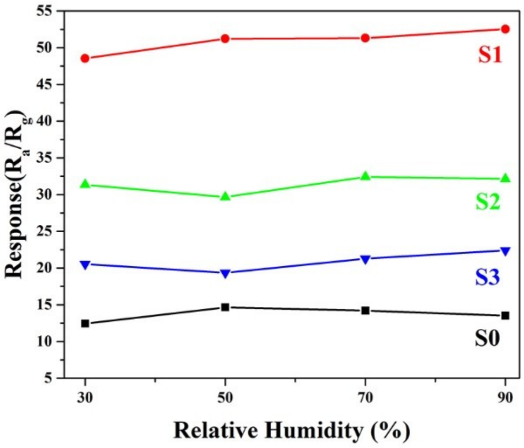

Fig.S5 Responses towards 100 ppm ethanol under different relative humidity.
